# Bridging the Mind and Body: Functional Neurologic Symptom Disorder Following Sexual Trauma in an Adolescent Girl

**DOI:** 10.7759/cureus.88181

**Published:** 2025-07-17

**Authors:** Ariel E Sosa Gomez, Daniel Gutierrez

**Affiliations:** 1 Psychiatry, The University of Texas Rio Grande Valley, Harlingen, USA; 2 Psychiatry, Tropical Texas Behavioral Health, Edinburg, USA

**Keywords:** cognitive behavioural therapy (cbt), fluoxetine depression, functional neurological disorder, major depression disorder, post traumatic stress disorder (ptsd)

## Abstract

Functional neurologic symptom disorder (FNSD) is a multifaceted condition that can present with neurologic symptoms inconsistent with recognized neurological disease, often in the context of psychosocial stressors, though these are not required for diagnosis. We report the case of a 12-year-old Hispanic female who developed episodes of limb paralysis, motor unresponsiveness, and self-injurious behaviors following a history of sexual abuse by a family member. Despite the initial absence of depressive symptoms, the patient later met criteria for major depressive disorder (MDD) and post-traumatic stress disorder (PTSD). Her neurological symptoms lacked identifiable medical findings, following unremarkable results from comprehensive blood work, a brain CT scan, MRIs, and an EEG. She was ultimately diagnosed with FNSD. Her care involved multiple psychiatric hospitalizations and trials of selective serotonin reuptake inhibitors (SSRIs), with partial response, complicated by psychosocial instability and poor adherence. Trauma-focused cognitive behavioral therapy (CBT-TF) was initiated and continued alongside pharmacologic treatment. At follow-up, the patient showed modest improvement in mood and functioning. There was a considerable reduction in the frequency and duration of her episodes, and her mood improved, from being dysthymic and socially withdrawn to becoming more engaged and euthymic, which led to increased participation in school and family activities. This case emphasizes the need for individualized, trauma-informed, and developmentally sensitive approaches to reduce extensive medical work-ups and optimize care in pediatric FNSD.

## Introduction

Functional neurologic symptom disorder (FNSD), formerly known as conversion disorder, presents significant challenges in pediatric and adolescent populations. This condition manifests with neurological-like symptoms without an identifiable physical cause and often affects individuals who have undergone substantial stress or trauma. The complexity of FNSD arises from its diverse symptomatology, which can include non-epileptic seizures, atypical gait, sensory disturbances, and even paralysis [1,2].

The diagnostic and management processes are complicated by the variability in symptoms. Conventional diagnostic tools, such as MRIs and EEGs, typically reveal no abnormalities, leading clinicians to rely on comprehensive clinical assessments that adhere to the criteria outlined in the Diagnostic and Statistical Manual of Mental Disorders, Fifth Edition (DSM-5). These evaluations focus on excluding physical etiologies, identifying psychological stressors, and recognizing atypical signs that do not conform to standard neurological or medical presentations [3].

Treatment for FNSD is inherently integrative, combining psychological and physical therapeutic interventions tailored to the individual’s unique needs and experiences. Cognitive-behavioral therapy (CBT) is commonly employed to address the psychological components, while physical therapy targets the somatic symptoms. Additionally, pharmacological interventions may be prescribed to manage comorbid conditions such as anxiety or depression, which frequently coexist in this population [4,5].

Despite advancements in understanding FNSD, significant gaps remain in elucidating the underlying mechanisms and in developing improved therapeutic modalities. Emerging research points to dysfunction within neuron-glial networks, chronic stress-system activation, and maladaptive predictive coding as contributing to symptom persistence in vulnerable children, particularly those with histories of trauma or insecure attachment. These findings highlight the importance of trauma-informed, biopsychosocial approaches that integrate neurological, psychological, and environmental considerations [6-8].

We present the case of a 12-year-old Hispanic female who experienced severe post-traumatic stress disorder (PTSD) and depression following a traumatic incident, and exhibited suicidal ideations and self-injurious behaviors. Her recovery was further complicated by physical manifestations such as episodic non-epileptic seizures and inconsistent sensory losses. These symptoms lacked correlation with identifiable medical pathologic complications, complicating the diagnostic process, and were ultimately attributed to FNSD. This case underscores the intricate relationship between psychological trauma and physical health in young patients, highlighting the imperative for a careful and nuanced approach to both diagnosis and treatment.

## Case presentation

This is the case of a 12-year-old Hispanic female who has been followed in our outpatient psychiatric service since May 2024. She was initially referred for skills training due to mild self-injurious behavior (SIB), which was not accompanied by clinically significant anxiety or depressive symptoms at that time. Her psychiatric history was unremarkable, with no prior mental health treatment or history of neurodevelopmental disorders.

In September 2024, the patient was referred to our clinic by Child Protective Services (CPS) following the disclosure of sexual abuse by a family member, which occurred in August 2023. Notably, the patient had not previously spoken about the abuse until the topic surfaced during a school writing class, prompting the teacher to contact CPS. During the initial psychiatric evaluation, the patient reported emotional dysregulation, feelings of shame, and fear of judgment surrounding the disclosure of the abuse. She also endorsed engaging in self-injurious behaviors, specifically superficial cutting of her forearms with a pencil sharpener, occurring three to four times per week. Despite this, she consistently denied suicidal ideation, plans, or intent.

At that time, she did not meet diagnostic criteria for a specific depressive disorder. Trauma-focused cognitive behavioral therapy (CBT-TF) was recommended, with follow-up scheduled in two to three months. Selective serotonin reuptake inhibitor (SSRI) treatment was to be considered if symptoms persisted.

In December 2024, she returned for follow-up and reported no worsening of depressive symptoms but described significant anxiety, particularly in crowded settings and classrooms. She reported episodes of overwhelming anxiety that led her to abruptly leave class. Approximately two weeks later, in January 2025, she experienced her first psychiatric hospitalization after engaging in SIB in a school bathroom. During this inpatient stay, she was started on escitalopram 5 mg daily and trazodone 50 mg at bedtime. Trazodone was quickly discontinued by her family due to excessive sedation. Shortly after initiating escitalopram, the patient was brought to the emergency department by her family with complaints of blurry vision, dizziness, and difficulty concentrating. Her primary care provider discontinued both medications. Due to persistent symptoms of depression and anxiety, she was then started on fluoxetine 10 mg daily by one of our clinic’s mid-level providers.

In February 2025, she presented for follow-up and reported a second psychiatric hospitalization that had occurred two weeks after her previous appointment. She was admitted due to crying spells and thoughts of self-injury while at school. Her parents noted ongoing depressive symptoms, frequently triggered by peer-related stressors. As a result, they chose to keep her home from school. Although an increase in fluoxetine to 20 mg daily was recommended, the family declined.

In March 2025, she returned following a third psychiatric hospitalization related to increased SIB and emergent suicidal ideation. She had become emotionally overwhelmed and frustrated. Upon discharge, she was prescribed fluoxetine 20 mg daily and aripiprazole 5 mg daily. However, her family did not administer the aripiprazole, and she continued on fluoxetine monotherapy. The family attributed this most recent crisis to the onset of menstruation. At this point, the patient’s care was formally transferred to our team for more intensive outpatient monitoring.

In April 2025, during our team’s initial evaluation, she presented as anxious and tearful, with daily crying episodes, pervasive feelings of hopelessness and worthlessness, and excessive guilt, particularly related to her history of trauma. She also exhibited disrupted sleep and energy, along with marked social withdrawal. She continued to engage in SIB and had experienced suicidal ideation without a plan, leading to a fourth psychiatric hospitalization in March 2025. During that admission, risperidone was initiated on top of fluoxetine 20mg daily; however, the family again declined to provide the medication after discharge. Our clinical team provided psychoeducation regarding her diagnosis and emphasized the importance of adherence to the psychopharmacological regimen to support her recovery and reduce symptom severity.

The patient reported limited engagement with trauma content in school-based therapy. She also described worsening academic performance and increased feelings of social inadequacy. Her parents reported an episode that resembled neurological dysfunction, during which the patient became unresponsive and paralyzed, appeared to have a flat affect, and was unable to answer questions, though she later reported being aware of her surroundings. The episode, which lasted approximately three hours, was initially witnessed by her parents and subsequently observed by healthcare providers after she was brought to the emergency department. Laboratory tests and neuroimaging, including a brain CT scan, were unremarkable. She was discharged with no additional medications aside from the previously prescribed fluoxetine 20 mg daily, as recommended by her psychiatrist. At that time, she was not experiencing suicidal ideation. A working diagnosis of major depressive disorder (MDD), PTSD, and possible FNSD, more specifically functional movement disorder (FMD), was made by our team. She was continued on fluoxetine and referred to a new trauma-focused counselor.

Approximately 10 days later, she returned for a post-hospitalization follow-up after a brief three-day psychiatric admission. This hospitalization was prompted by suicidal ideation with intent to hang herself following a peer conflict. She disclosed that her former best friend had distanced herself due to witnessing the patient’s episodes, which included sudden loss of motor function, collapsing to the ground, and apparent unresponsiveness. The patient described these episodes as episodes of neurological dysfunction during which she was aware of her environment but unable to respond. These episodes typically lasted about 15 minutes and were not associated with loss of consciousness. They appeared to be triggered by emotional distress, particularly related to traumatic memories.

During her May 2025 follow-up, the patient had not been re-hospitalized. She continued CBT-TF, and reported some improvement in depressive and anxiety symptoms. However, she continued to experience functional neurologic episodes, particularly in response to physical interactions with male peers, even when playful and non-sexual. She also disclosed an incident involving playful and regular physical contact by female classmates, which she interpreted as inappropriate and subsequently reported to school personnel and CPS. The patient provided limited detail about the incident, and further information was unavailable.

A neurological workup was completed, including EEG and neuroimaging, which ruled out any underlying neurological abnormalities contributing to her presentation. She was advised to continue CBT-TF and fluoxetine 20 mg daily. During this visit, the patient appeared detached, with minimal eye contact and limited verbal responses, often restricted to “yes” or “no.” Her mood was neutral, with restricted affect, and her thinking was concrete, characterized by limited capacity for abstraction. She denied active suicidal ideation but continued to engage in self-injurious behaviors. There were no signs of psychosis or perceptual disturbances.

In June 2025, the patient returned to the clinic with notable improvement. She was more open to engaging in conversation, displayed a brighter and euthymic affect, and her suicidal ideation and self-injurious behaviors had resolved. However, some residual symptoms persisted, including sleep disturbances and low motivation. Her functional neurologic episodes had also decreased in both frequency and duration, now occurring sporadically, less than once per week, and lasting approximately 10 to 15 minutes. She continued to deny any perceptual disturbances or delusional constructs.

## Discussion

This case illustrates the diagnostic and therapeutic challenges inherent in treating pediatric patients with FNSD, especially in the context of comorbid PTSD and MDD. Our patient, a 12-year-old Hispanic female, demonstrated a complex interplay between psychological trauma, emotional dysregulation, and recurrent episodes of apparent neurologic dysfunction, all of which were best conceptualized under the umbrella of FNSD.

According to the DSM-5, Text Revision (DSM-5-TR) [9], FNSD is characterized by one or more symptoms of altered voluntary motor or sensory function that are incompatible with recognized neurological or medical conditions. The diagnosis requires clinical findings that demonstrate clear evidence of internal inconsistency or incongruence with known pathophysiological mechanisms. Additionally, the symptom must cause significant distress or impairment in social, occupational, or other areas of functioning, and must not be better explained by another medical or mental disorder. In this case, the patient's presentation aligns closely with these criteria, particularly the inconsistency of neurological findings and the functional impairment associated with her episodes.

Diagnostic considerations for FNSD

The diagnosis of FNSD in this case is supported by several clinical features that are incongruent with known neurological pathologies. The patient exhibited episodic limb paralysis, sudden unresponsiveness, and collapsed posture without loss of consciousness, postictal confusion, or EEG-confirmed seizure activity, features that are more consistent with psychogenic non-epileptic seizures (PNES) or functional movement phenomena rather than true epileptic or structural neurological disorders [1,3,6]. Additionally, these episodes were often precipitated by identifiable psychological stressors, such as peer rejection or traumatic reminders, and resolved spontaneously without medical intervention. These characteristics are commonly reported in FNSD and are considered red flags for a functional etiology [2,3]. The absence of positive neurological findings during emergency department evaluations, including unremarkable lab results and lack of acute neurological signs, further supports a functional diagnosis. Nonetheless, recognizing the risk of misdiagnosis and the importance of excluding organic pathology, we strongly recommended neuroimaging (e.g., MRI) and EEG evaluation as part of her ongoing assessment, which later demonstrated unremarkable results [6,7].

Psychological trauma and the body

There is growing evidence linking early life trauma to the development of FNSD, particularly in pediatric populations. The somatization of emotional distress, especially among children and adolescents with limited psychological resources, is a well-documented mechanism by which unresolved trauma can manifest physically [2,4]. In our patient, the onset of neurologic-like symptoms closely followed the disclosure of sexual trauma, a time during which she experienced escalating anxiety, shame, and self-injurious behavior. This pattern aligns with prior studies noting that trauma-related disorders, particularly PTSD, often co-occur with or precede the development of functional neurological symptoms [1,4,5]. Notably, the patient’s dissociative-like presentations (unresponsiveness, transient paralysis, emotional numbing) appeared to function as ineffective coping strategies in response to distressing emotional or interpersonal stress. This dissociative quality is supported by phenomenological research that describes such episodes as protective mechanisms against overwhelming internal or external stimuli [5].

Cognitive development and neuropsychological profile

In assessing the patient’s cognitive and behavioral profile, it became evident that her responses were often concrete, literal, and lacked abstraction. She showed difficulty engaging in reflective or hypothetical reasoning and provided short, affirmational replies (e.g., “yes” or “no”), even to open-ended questions. These characteristics are consistent with Piaget’s Concrete Operational Stage of Cognitive Development, typically observed in children aged seven to 11 years [10]. At this developmental stage, children begin to think logically about concrete events but struggle with abstract, hypothetical, or deductive reasoning. Our patient’s limited ability to engage in abstract discussion about her trauma, her avoidance of emotional content in therapy, and her difficulty in identifying complex social dynamics suggest she may still be operating primarily at the concrete operational level. This aligns with her observable difficulty in linking her emotional states to past events or in projecting long-term consequences, both of which are critical for insight-driven therapy [4,5]. Such developmental constraints may partially explain her limited progress in school-based therapy and may highlight the need for tailored psychotherapeutic approaches, including developmentally sensitive trauma-focused CBT that incorporates concrete, structured activities, visual aids, and body-based regulation strategies.

Comorbid psychiatric disorders and treatment implications

Our patient’s clinical course was further complicated by recurrent depressive episodes, suicidal ideation, and emotional dysregulation, resulting in four psychiatric hospitalizations within six months. These comorbidities necessitated pharmacological intervention. While fluoxetine has been relatively well-tolerated, previous trials of escitalopram and antipsychotic augmentation (trazodone, risperidone, aripiprazole) were discontinued due to adverse effects or parental non-adherence. Escitalopram was associated with nausea and headaches, and the antipsychotics were discontinued by the parents due to concerns about side effects and long-term use. Notably, fluoxetine led to significant improvement in the patient’s affective symptoms, with a transition from a dysthymic to a euthymic mood, increased energy and activity levels, reduced feelings of hopelessness and worthlessness, and complete resolution of suicidal ideation. The concurrent use of trauma-focused psychotherapy was also instrumental in mitigating depressive symptoms by facilitating the processing and discussion of her trauma history. These challenges and improvements highlight both the complexity of pharmacologic management in pediatric functional disorders and the critical importance of psychoeducation, family engagement, and integrated therapeutic interventions in treatment planning. Given her limited response to previous outpatient therapy and ongoing safety concerns, referral to a wraparound program with daily monitoring and multidisciplinary care was also considered.

Recommendations 

The working diagnosis of FNSD remains the most clinically consistent explanation for the patient’s episodic neurologic symptoms, particularly in light of the identified psychogenic triggers, unremarkable medical findings, and the fluctuating course of her symptoms. To strengthen diagnostic certainty and optimize treatment outcomes, we recommend a comprehensive, multidisciplinary approach. This should include a neurological workup, including EEG and brain MRI, to rule out epileptiform activity or structural abnormalities; a comprehensive neuropsychological evaluation to assess for subtle neurodevelopmental deficits, learning disorders, or executive dysfunction that may affect her ability to engage in therapy; and the continuation of CBT-TF, ideally incorporating somatic, narrative, and creative modalities tailored to developmentally concrete youth. Additionally, family therapy and psychoeducation should be prioritized to improve medication adherence, enhance parental understanding of FNSD, and reduce environmental stressors or reinforcing patterns both at home and in the school setting.

## Conclusions

This case highlights the complex interplay between psychological trauma, mood disorders, and functional neurologic symptoms in a pediatric patient. The patient’s episodic neurological events, occurring alongside significant trauma and psychosocial stressors, supported a working diagnosis of FNSD. A comprehensive neurological workup, including neuroimaging and clinical observation, revealed no evidence of organic pathology. Over time, the patient demonstrated meaningful improvement with CBT-TF and fluoxetine, which helped stabilize mood and reduce the frequency of functional episodes, further supporting the functional diagnosis.

Cultural stigma, developmental limitations in emotional expression, and contextual stressors such as family conflict and academic difficulties likely influenced symptom expression and treatment engagement. Early access to trauma-informed psychotherapy, family psychoeducation, and multidisciplinary care played a critical role in the patient’s clinical progress and remain essential components in managing FNSD in pediatric populations.
